# Body Mass Index, Cardiometabolic Treatment Patterns and Multifactorial Risk‐Factor Control in Chinese Adults With Type 2 Diabetes: A Multicenter Cross‐Sectional Study

**DOI:** 10.1111/1753-0407.70296

**Published:** 2026-11-03

**Authors:** Jingya Yan, Wenjia Yang, Xiaoling Cai, Ming Liu, Yanbing Li, Qiuhe Ji, Bo Feng, Nanwei Tong, Linong Ji

**Affiliations:** ^1^ Department of Endocrinology Peking University People's Hospital Beijing China; ^2^ Beijing Key Laboratory of Innovative Drug and Device Translation in Endocrine and Metabolic Diseases Beijing China; ^3^ Department of Endocrinology and Metabolism Tianjin Medical University General Hospital Tianjin China; ^4^ Department of Endocrinology and Diabetes Center The First Affiliated Hospital of Sun Yat‐Sen University Guangzhou China; ^5^ Department of Endocrinology Xi'an International Medical Center Hospital Xi'an China; ^6^ Department of Endocrinology Shanghai East Hospital, Tongji University Shanghai China; ^7^ Department of Endocrinology and Metabolism West China Hospital, Sichuan University Chengdu China; ^8^ Peking University Diabetes Center Beijing China

**Keywords:** body mass index, cardiovascular risk, China, multiple imputation, Type 2 diabetes

## Abstract

**Background:**

To examine medication use and simultaneous achievement of glycemic, blood pressure and lipid targets across body mass index (BMI) categories in Chinese adults with Type 2 diabetes.

**Methods:**

This BMI‐focused secondary cross‐sectional analysis included 8590 adults with evaluable BMI recruited from 60 hospitals in the iCaReMe China Registry. The primary comparison was obesity versus normal weight for simultaneous achievement of HbA1c < 7.0%, blood pressure < 130/80 mmHg and LDL cholesterol < 2.6 mmol/L. Missing data were handled by multiple imputation (20 datasets; 20 iterations per chain), and associations were estimated with center‐clustered generalized estimating equations (GEE).

**Results:**

The cohort comprised 99 (1.2%) adults with underweight, 2725 (31.7%) normal weight, 3663 (42.6%) overweight, and 2103 (24.5%) obesity. Among 6683 with all three observed components, 332 (5.0%) achieved the composite. Compared with normal weight, the pooled adjusted OR for obesity was 0.55 (95% CI 0.39–0.78); each 1‐kg/m^2^ higher BMI was associated with an OR of 0.93 (0.90–0.96). For obesity, component‐specific ORs were 0.72 (0.61–0.85) for HbA1c, 0.47 (0.40–0.55) for blood pressure and 1.05 (0.91–1.22) for LDL‐C. Sensitivity analyses supported the composite finding, with no evidence of nonlinearity (*p* = 0.904).

**Conclusions:**

Obesity was associated with lower odds of simultaneous target attainment, with component‐specific associations for glycemia and blood pressure but not LDL‐C. GLP‐1 receptor agonists and SGLT2 inhibitors were used more often at higher BMI; these prescribing patterns do not establish treatment effectiveness.

**Trial Registration:** ChiCTR2300073764.

## Highlights

Among 8590 Chinese adults with Type 2 diabetes, only 5% of those with all three components measured simultaneously met the HbA1c, blood pressure, and LDL‐C targets. Obesity was associated with 45% lower odds of meeting all three targets, with lower odds of HbA1c and blood pressure attainment but no independent association with LDL‐C attainment. Recorded use of GLP‐1 receptor agonists and SGLT2 inhibitors increased across BMI categories.

## Graphical abstract

In this secondary analysis of 8590 Chinese adults with Type 2 diabetes, obesity was associated with lower odds of simultaneous glycemic, blood‐pressure, and lipid control than normal weight (adjusted OR 0.55; 95% CI 0.39–0.78). GLP‐1 receptor agonist and SGLT2 inhibitor use increased across BMI categories.

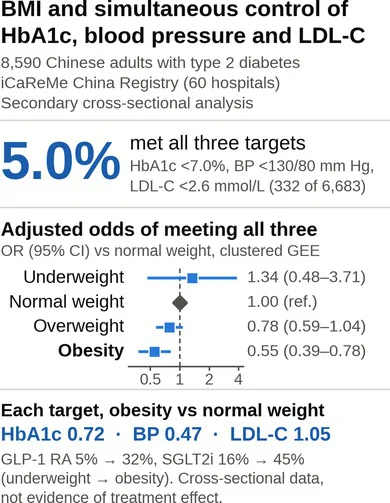

**Abbreviations**
ACS: acute coronary syndrome
ADA: American Diabetes Association
AF: atrial fibrillation
ALT: alanine aminotransferase
ARB: angiotensin receptor blocker
ASCVD: atherosclerotic cardiovascular disease
BMI: body mass index
BP: blood pressure
CCB: calcium channel blocker
CI: confidence interval
CKD: chronic kidney disease
CVD: cardiovascular disease
DBP: diastolic blood pressure
DPP‐4: dipeptidyl peptidase‐4
EASD: European Association for the Study of Diabetes
eGFR: estimated glomerular filtration rate
ESC: European Society of Cardiology
GLP‐1 RA: glucagon‐like peptide‐1 receptor agonist
HbA1c: glycated hemoglobin
HDL‐C: high‐density lipoprotein cholesterol
ICH‐GCP: International Council for Harmonization—Good Clinical Practice
IQR: interquartile range
LDL‐C: low‐density lipoprotein cholesterol
MI: myocardial infarction
MICE: multiple imputation by chained equations
OR: odds ratio
SBP: systolic blood pressure
SD: standard deviation
SGLT2: sodium‐glucose cotransporter‐2
T2D: Type 2 diabetes
UACR: urine albumin‐to‐creatinine ratio

## 1 Introduction

Type 2 diabetes affects more than 100 million adults in China [1], and its cardiovascular and renal complications have made simultaneous control of multiple risk factors a central goal of diabetes care [2]. Current guidelines recommend concurrent management of glycemia, blood pressure, and LDL cholesterol, together with glucagon‐like peptide‐1 receptor agonists and sodium‐glucose cotransporter‐2 inhibitors with proven cardiovascular or renal benefit [3, 4, 5] for patients with established cardiovascular or kidney disease [6, 7]. In China, however, achievement of the composite 3B target—HbA1c < 7.0%, blood pressure < 130/80 mmHg, and LDL‐C < 2.6 mmol/L—remains uncommon [8]. Understanding how this gap varies across common clinical phenotypes may help identify groups in whom comprehensive risk‐factor management needs greater attention.

Body mass index (BMI) is a readily available marker of this clinical heterogeneity. East Asian adults can develop Type 2 diabetes at a relatively low BMI, often with prominent β‐cell dysfunction, whereas higher BMI is associated with insulin resistance, hypertension, and atherogenic dyslipidemia [9, 10, 11, 12]. People with underweight may also have substantial vascular and nutritional risk [13, 14, 15, 16]. Whether target attainment varies across the BMI distribution, and in what shape, is therefore an empirical question. BMI categories are useful for clinical description, but continuous modeling retains within‐category information and can show departures from linearity without assuming an abrupt change at category boundaries.

Use of GLP‐1 receptor agonists and SGLT2 inhibitors has expanded in China [17], but contemporary data on how these agents are used alongside statins and antihypertensive drugs across BMI groups are limited. The iCaReMe design paper described the registry [18], and earlier conference and full reports described aggregate risk‐factor control in the 9000‐person diabetes cohort [8, 19]. Two additional conference abstracts described glucose‐lowering treatment patterns [20] and factors associated with cardiovascular disease [21] in the same cohort. Using the same baseline cohort, the present secondary analysis examines BMI‐specific differences in standardized 3B and component target attainment, models BMI continuously to assess the shape of these associations, and describes medication use by BMI category.

We therefore analyzed baseline data from adults with Type 2 diabetes enrolled in the iCaReMe China Registry. The primary comparison of this secondary analysis was obesity versus normal weight for the standardized 3B target. Other BMI‐category contrasts, the individual components, medication patterns, continuous BMI, and sensitivity analyses were secondary or exploratory.

## 2 Materials and Methods

### 2.1 Study Design and Setting

This secondary cross‐sectional study used baseline data collected from July 2023 to March 2024 in the Type 2 diabetes cohort of the iCaReMe China Registry (ChiCTR2300073764) [18]. The parent registry is a prospective observational study, and the 9000‐person Type 2 diabetes cohort was recruited from 60 hospitals, as reported previously [8]. The analytic dataset contained 61 recruitment‐center codes, which could not be mapped one‐to‐one to hospitals from the files available to us. All 61 codes were used as GEE clusters; the smallest code contained four participants, and a sensitivity analysis excluding it left 60 clusters.

### 2.2 Participants and Analytic Sample

Participants were adults with an existing clinical diagnosis of Type 2 diabetes recorded at the enrolling site [18]. Of 9000 enrolled records, 408 lacked height or weight and two had a recorded age below 18 years. The primary analytic cohort therefore comprised 8590 adults with calculable BMI. The analysis used the same recruitment period and diabetes cohort as the earlier aggregate report [8], but addressed a BMI‐focused question with different exposure definitions and statistical models (Table S1). Characteristics of BMI‐evaluable and BMI‐unavailable records were compared (Table S2), and selection‐probability weighting was used as a sensitivity analysis.

### 2.3 Data Collection and Measurements

Baseline data included demographic and socioeconomic characteristics, smoking, alcohol consumption, and physical activity. Anthropometric measurements included height, weight, waist circumference, and blood pressure. Laboratory measurements included HbA1c, fasting glucose, LDL cholesterol (LDL‐C), HDL cholesterol, triglycerides, estimated glomerular filtration rate, urine albumin‐to‐creatinine ratio, and alanine aminotransferase. Medication classes were recorded at the baseline visit. Standardized macrovascular and microvascular history variables were derived from the registry source fields; coding rules and availability are detailed in the Supplementary Methods.

The primary outcome was the standardized 3B target: simultaneous attainment of HbA1c < 7.0%, systolic blood pressure < 130 mmHg and diastolic blood pressure < 80 mmHg, and LDL‐C < 2.6 mmol/L. The uniform LDL‐C threshold was used to apply a common outcome definition across BMI groups. It serves as a standardized quality‐of‐care measure, rather than an individualized treatment goal; guideline‐recommended LDL‐C goals vary by cardiovascular risk [6, 7]. For observed‐data summaries, the composite was achieved only when all three components were observed and met; it was a known failure when any observed component failed; and it was indeterminate only when at least one component was missing and none of the observed components failed. Within each imputed dataset, component targets were derived passively from imputed continuous measurements and then combined into the composite.

Component‐specific descriptive proportions used the available denominator for that component. Of 8590 adults, 6683 had all three observed components, 1557 had incomplete measurements but at least one observed failure, and 350 had an indeterminate observed composite. In the primary analysis, missing components were multiply imputed rather than coded as failure. Missingness was summarized overall, by BMI category and by center (Tables S3 and S4), and participants with complete and incomplete 3B components were compared (Table S5).

### 2.4 Main Independent Variable: BMI

BMI was calculated as weight in kilograms divided by height in meters squared. Chinese‐specific cutoffs were used: underweight (< 18.5 kg/m^2^), normal weight (18.5 to < 24.0 kg/m^2^), overweight (24.0 to < 28.0 kg/m^2^) and obesity (≥ 28.0 kg/m^2^) [22]. Waist‐to‐height ratio (WHtR) was used in a central‐adiposity sensitivity model.

### 2.5 Statistical Analysis

Continuous variables are presented as median (interquartile range) and categorical variables as *n*/*N* (%), with evaluable denominators stated. Group comparisons used Kruskal–Wallis tests for continuous variables and *χ* ^2^ or Fisher exact tests for categorical variables, as appropriate. Descriptive estimates were based on observed data. The obesity‐versus‐normal‐weight comparison for the composite target was treated as the primary comparison of this secondary analysis; other *p* values are nominal and were not adjusted for multiplicity.

The primary population‐averaged association was estimated with logistic GEE using an exchangeable working correlation and robust sandwich standard errors, clustered by recruitment‐center code. GEE was chosen because the question concerned the population‐averaged association across centers rather than center‐specific effects. Models adjusted for age, sex, diabetes duration, smoking, alcohol use, education, physical activity, and standardized macrovascular history; normal weight was the reference. Pooled inferences used Rubin's rules with small‐sample finite‐cluster degrees of freedom.

Continuous BMI associations with the composite and individual component targets were modeled with restricted cubic splines using knots at the 5th, 35th, 65th, and 95th percentiles (20.28, 24.17, 26.84, and 32.70 kg/m^2^), with BMI 24 kg/m^2^ as the reference. D1 joint tests assessed the overall BMI contribution and nonlinearity beyond the linear term. Composite‐outcome sensitivity analyses used three and five knots and repeated the primary spline after trimming the 1st and 99th BMI percentiles.

Sensitivity analyses included complete‐case GEE; an independence working correlation; exclusion of the four‐participant center; selection‐probability weighting for availability of BMI; a linear BMI model trimmed at the 1st and 99th percentiles; an exploratory model containing BMI and WHtR; exploratory adjustment for insulin, metformin, SGLT2 inhibitor, GLP‐1 receptor agonist, antihypertensive and statin use; and two delta‐adjusted missing‐not‐at‐random scenarios. Medication‐adjusted models were exploratory because treatment may reflect disease severity, access, and prior response.

Missing covariates and continuous outcome components were handled by chained‐equations multiple imputation. Twenty independent completed datasets were generated in four batches of five using fixed random seeds, with 20 iterations per chain. Predictive mean matching with five donors was used for continuous variables and stochastic logistic or multinomial regression for categorical variables. BMI, center code and observed values were not altered. Trace summaries, R‐hat values, Monte Carlo precision, model convergence, and data‐integrity checks are reported in the Table S6, Figures S1 and S2. Analyses used Python 3.12 implementations of the specified imputation, GEE, and Rubin‐pooling procedures.

## 3 Results

### 3.1 Study Population and BMI Distribution

Of 9000 enrolled records, 408 were excluded because height or weight was unavailable and two because recorded age was below 18 years, leaving 8590 adults. There were 99 (1.2%) participants with underweight, 2725 (31.7%) with normal weight, 3663 (42.6%) with overweight, and 2103 (24.5%) with obesity (Figure S3).

Participants with obesity were younger than those with normal weight (median 50.0 vs. 58.0 years) and had shorter diabetes duration (3.4 vs. 6.7 years). Waist circumference, WHtR, blood pressure, and triglycerides increased across BMI categories (Table 1).

**TABLE 1:** Observed clinical characteristics by BMI category.

Characteristic	Underweight (*n* = 99)	Normal weight (*n* = 2725)	Overweight (*n* = 3663)	Obesity (*n* = 2103)	*p*
Male sex, *n*/*N* (%)	49/99 (49.5%)	1489/2725 (54.6%)	2354/3663 (64.3%)	1341/2103 (63.8%)	< 0.001
Age, years	60.0 (51.0–66.5)	58.0 (50.0–65.0)	56.0 (47.0–63.0)	50.0 (39.0–59.0)	< 0.001
Diabetes duration, years	5.5 (0.6–10.7)	6.7 (1.6–13.1)	5.8 (1.2–12.1)	3.4 (0.3–9.6)	< 0.001
Waist circumference, cm	75.0 (69.0–79.5)	84.0 (80.0–90.0)	92.0 (86.2–97.0)	102.0 (96.0–108.5)	< 0.001
Waist‐to‐height ratio	0.45 (0.42–0.48)	0.51 (0.48–0.54)	0.55 (0.52–0.58)	0.60 (0.57–0.64)	< 0.001
Systolic BP, mm Hg	120.0 (112.0–138.0)	127.0 (120.0–138.0)	130.0 (120.0–140.0)	131.0 (122.0–143.0)	< 0.001
Diastolic BP, mm Hg	75.0 (69.0–83.0)	80.0 (72.0–85.0)	80.0 (75.0–88.0)	83.0 (78.0–90.0)	< 0.001
HbA1c, %	8.2 (7.0–10.6)	7.8 (6.6–10.0)	8.0 (6.8–9.8)	8.4 (7.0–10.3)	< 0.001
Fasting glucose, mmol/L	7.6 (6.3–9.9)	7.6 (6.2–9.9)	7.9 (6.5–10.3)	8.4 (6.8–11.2)	< 0.001
LDL‐C, mmol/L	2.69 (2.04–3.24)	2.71 (2.08–3.39)	2.77 (2.12–3.44)	2.84 (2.20–3.49)	0.004
HDL‐C, mmol/L	1.34 (1.02–1.65)	1.19 (0.99–1.42)	1.09 (0.92–1.28)	1.04 (0.88–1.22)	< 0.001
Triglycerides, mmol/L	1.01 (0.74–1.35)	1.33 (0.94–2.08)	1.70 (1.18–2.59)	2.00 (1.36–3.23)	< 0.001
eGFR, mL/min/1.73 m^2^	103.2 (93.1–112.6)	99.3 (89.0–108.5)	100.0 (88.1–110.6)	105.0 (93.0–116.0)	< 0.001
UACR, mg/g	13.4 (8.4–62.4)	14.1 (6.8–33.2)	15.0 (7.3–39.3)	17.7 (8.0–51.4)	< 0.001
ALT, U/L	17.4 (10.1–26.0)	19.0 (14.0–27.0)	22.0 (16.0–33.0)	28.9 (19.0–47.0)	< 0.001
Achieved 3B target, *n*/*N* (%)	3/72 (4.2%)	140/2098 (6.7%)	139/2844 (4.9%)	50/1669 (3.0%)	< 0.001

*Note:* Values are median (interquartile range) unless otherwise stated; categorical variables are *n*/*N* (%). Denominators reflect available observed data. The composite 3B row is restricted to participants with all three observed components. *p* values compare the four BMI categories.

Abbreviations: ALT, alanine aminotransferase; BP, blood pressure; eGFR, estimated glomerular filtration rate; HbA1c, glycated hemoglobin; HDL‐C, high‐density lipoprotein cholesterol; LDL‐C, low‐density lipoprotein cholesterol; UACR, urine albumin‐to‐creatinine ratio.

### 3.2 Cardiometabolic Target Achievement by BMI Category

Target achievement varied across BMI categories (Figure 1). HbA1c target attainment was 21.6% in underweight, 33.0% in normal weight, 28.2% in overweight, and 23.9% in obesity. Corresponding blood‐pressure target attainment was 48.4%, 35.8%, 25.9%, and 20.0%, and LDL‐C target attainment was 45.6%, 45.1%, 42.9%, and 40.3%.

**FIGURE 1:**
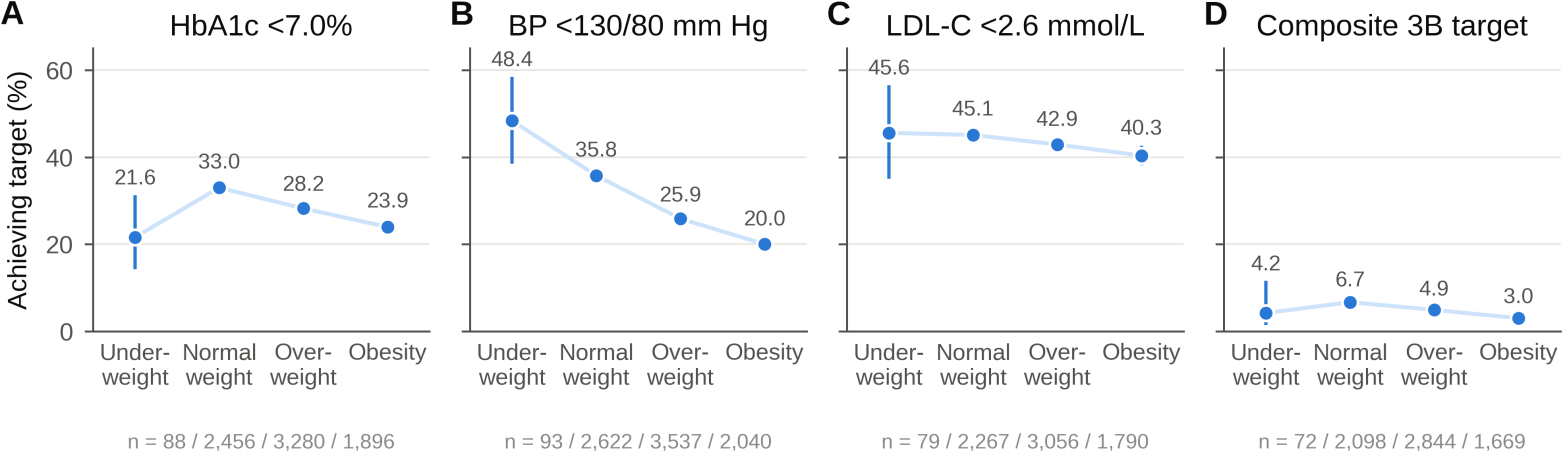
Observed target achievement by BMI category. Points show the observed proportion of participants achieving each target and vertical lines the 95% Wilson confidence interval, among participants with the relevant observed component (denominators under each panel: Underweight/normal weight/overweight/obesity). Connecting lines are visual guides only. (A) HbA1c < 7.0%; (B) blood pressure < 130/80 mmHg; (C) LDL‐C < 2.6 mmol/L; (D) composite 3B target, restricted to participants with all three observed components (*n* = 6683).

Among 6683 participants with all three observed components, 332 (5.0%) achieved the composite: 3/72 (4.2%) with underweight, 140/2098 (6.7%) with normal weight, 139/2844 (4.9%) with overweight, and 50/1669 (3.0%) with obesity. The observed‐data outcome‐status breakdown is shown in Table S7.

### 3.3 Standardized Vascular‐History Covariates

Standardized macrovascular history was recorded in 93 participants with underweight, 2477 with normal weight, 3403 with overweight and 1934 with obesity; the corresponding observed prevalences were 26.9%, 20.3%, 23.2%, and 18.6%. Because availability varied, these values are reported with explicit denominators in Table S8.

Observed standardized microvascular history was present in 35.8%, 24.5%, 25.8%, and 24.1% of evaluable participants in the underweight, normal‐weight, overweight, and obesity groups, respectively. These history variables were used as clinical descriptors; only macrovascular history was included in the primary adjustment set.

Documented hypertension was most frequent in obesity (996/2027; 49.1%), compared with 29/96 (30.2%) in underweight. Documented history may reflect completeness of recording as well as true prevalence.

### 3.4 Adjusted Associations of BMI Categories With the Composite Target

In the primary multiply imputed GEE, obesity was associated with lower odds of composite 3B target achievement than normal weight (OR 0.55, 95% CI 0.39–0.78; *p* = 0.001). Estimates were 0.78 (0.59–1.04; *p* = 0.090) for overweight and 1.34 (0.48–3.71; *p* = 0.568) for underweight (Figure 2; Table S9). The complete‐case obesity estimate was 0.50 (0.36–0.70). Excluding the four‐record center produced an obesity OR of 0.55 (0.39–0.78), and selection‐probability weighting with an independence working correlation produced 0.53 (0.36–0.77) (Table S10). The underweight estimate was imprecise and was treated as exploratory.

**FIGURE 2:**
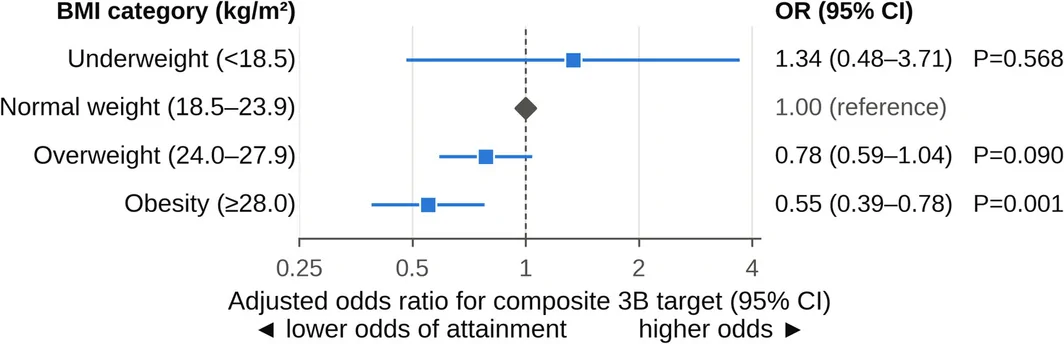
Center‐clustered adjusted association of BMI categories with composite 3B target achievement. Squares indicate pooled adjusted odds ratios and horizontal lines 95% confidence intervals from exchangeable‐correlation logistic GEE across 20 multiply imputed datasets (20 iterations per chain), clustered by recruitment‐center code with robust standard errors. Models adjusted for age, sex, diabetes duration, smoking, alcohol use, education, physical activity, and standardized macrovascular history; normal weight (diamond) was the reference. Obesity versus normal weight was the primary comparison of this secondary analysis; other *p* values are nominal.

### 3.5 Medication Treatment Patterns Across BMI Categories

Glucose‐lowering regimens differed across BMI groups (Figure 3; Table S11). Insulin use was highest in underweight (68.7%) and lowest in obesity (35.7%). GLP‐1 receptor agonist use increased from 5.1% in underweight to 32.1% in obesity, and SGLT2 inhibitor use increased from 16.2% to 45.4%.

**FIGURE 3:**
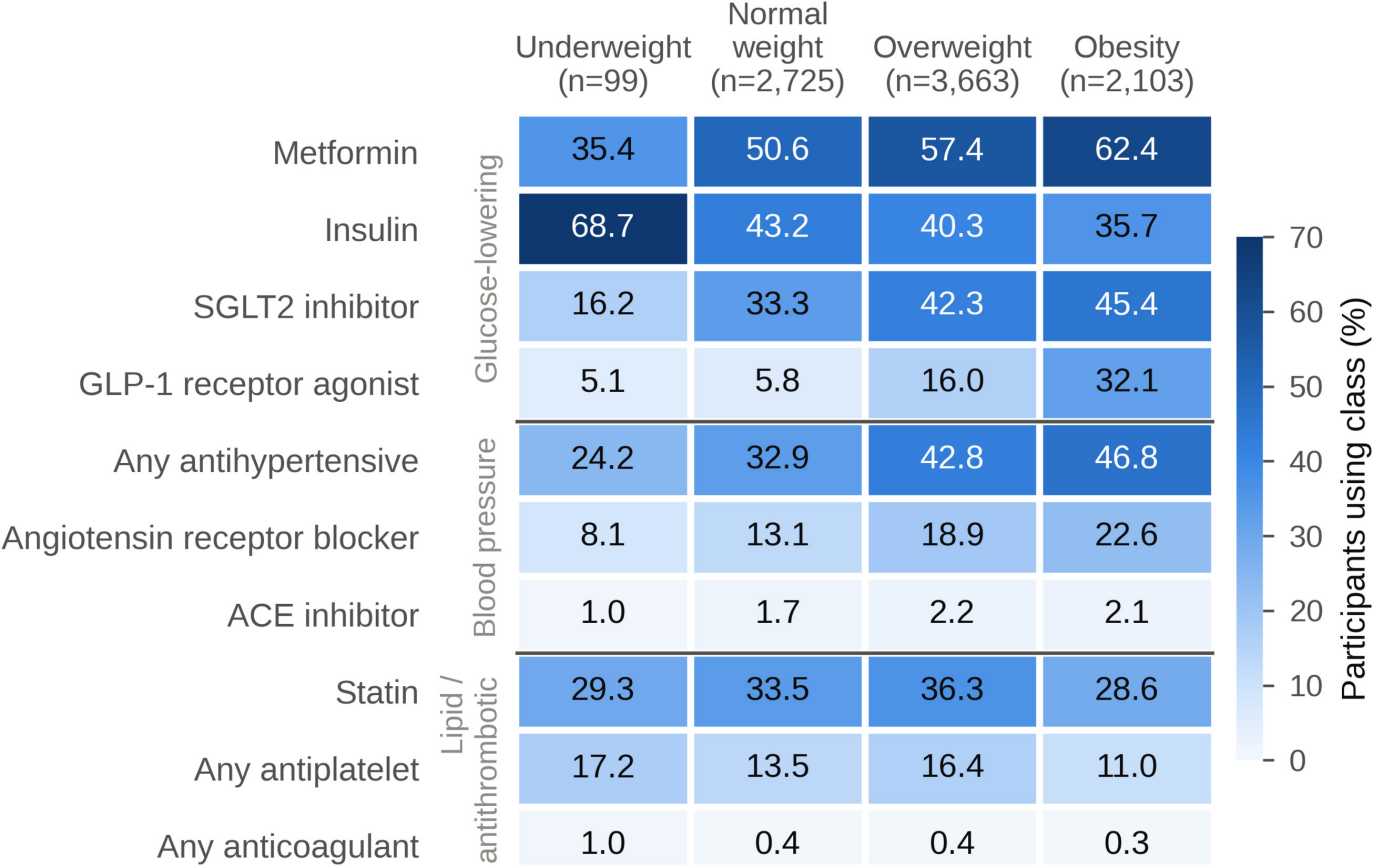
Baseline medication use by BMI category. Cells show the observed percentage of participants with recorded use of each of the 10 medication classes consistently available in the adult analytic dataset; denominators are the full adult BMI groups (*n* in column headers). Rows are grouped as glucose‐lowering, blood‐pressure‐lowering, and lipid‐lowering/antithrombotic classes. Medication classes are not mutually exclusive.

Any antihypertensive use increased from 24.2% in underweight to 46.8% in obesity. Statin use was 29.3%, 33.5%, 36.3%, and 28.6% across the four BMI categories. Denominators were the full BMI groups; indication, dose, duration, adherence, and contraindication data were not available.

### 3.6 Component‐Specific and Continuous BMI Associations

Compared with normal weight, obesity was associated with lower odds of HbA1c target attainment (OR 0.72, 95% CI 0.61–0.85) and blood‐pressure target attainment (OR 0.47, 95% CI 0.40–0.55). There was no evidence of an independent association with LDL‐C target attainment (OR 1.05, 95% CI 0.91–1.22) (Table S12; Figure S4).

BMI was associated with the composite target overall (D1 *p* < 0.001), without evidence that a spline improved on a linear term (*p* for nonlinearity = 0.904; Figure 4; Table S13 and Figure S5). Nonlinearity remained unsupported with three knots (*p* = 0.751), five knots (*p* = 0.851) and after trimming the 1st and 99th BMI percentiles (*p* = 0.680; Table S14). In the linear model, each 1‐kg/m^2^ higher BMI was associated with an OR of 0.93 (95% CI 0.90–0.96; *p* < 0.001). Nonlinear associations were detected for HbA1c target attainment (*p* = 0.001) and blood‐pressure target attainment (*p* = 0.021), but not LDL‐C target attainment (*p* = 0.382; overall *p* = 0.556).

**FIGURE 4:**
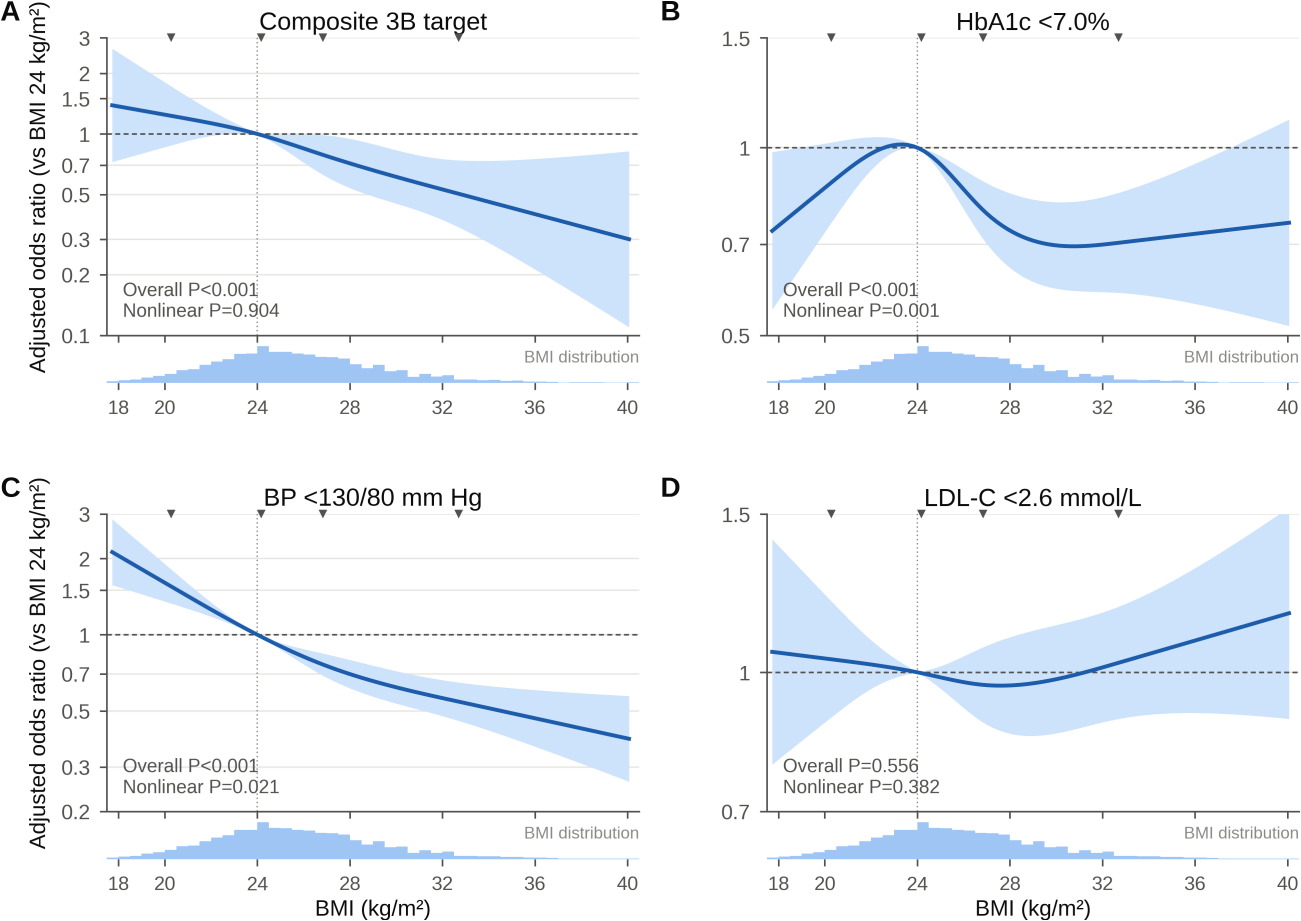
Adjusted associations of continuous BMI with target attainment using restricted cubic splines. Curves show pooled adjusted odds ratios (log scale) and shaded bands pointwise 95% confidence intervals relative to BMI 24 kg/m^2^ (vertical dotted line), from center‐clustered GEE models pooled across 20 imputed datasets with the covariates of Figure 2. Triangles mark the knots (5th, 35th, 65th, and 95th percentiles: 20.28, 24.17, 26.84, and 32.70 kg/m^2^). Curves span the 0.5th to 99.5th BMI percentiles, and the histogram strip beneath each panel shows the observed BMI distribution of the analytic cohort (0.5 kg/m^2^ bins). D1 joint tests in each panel compare the spline with a model omitting BMI (overall) and with a linear BMI term (nonlinear). (A) Composite 3B target; (B) HbA1c < 7.0%; (C) blood pressure < 130/80 mmHg; (D) LDL‐C < 2.6 mmol/L.

Results were consistent under an independence working correlation and both delta‐adjusted missing‐not‐at‐random scenarios (Table S9), and the linear BMI estimate was unchanged after trimming the 1st and 99th BMI percentiles (OR 0.93 per 1 kg/m^2^, 95% CI 0.90–0.97; *n* = 8419). In the exploratory model containing both BMI and WHtR, the ORs were 0.96 (95% CI 0.91–1.00; *p* = 0.061) per 1‐kg/m^2^ BMI and 0.77 (0.56–1.06; *p* = 0.109) per 0.1 higher WHtR. Exploratory medication adjustment attenuated but did not remove the obesity association (OR 0.60, 0.43–0.85).

## 4 Discussion

In this contemporary multicenter registry, only 5.0% of participants with complete observed components achieved all three cardiometabolic targets. After multiple imputation, covariate adjustment and center clustering, obesity was associated with approximately 45% lower odds of composite attainment than normal weight. The component analyses identified lower odds of glycemic and blood‐pressure target attainment, with no independent association for LDL‐C attainment. The composite estimate was consistent across complete‐case, correlation‐structure, and missing‐not‐at‐random sensitivity analyses.

Earlier iCaReMe reports described aggregate risk‐factor control, glucose‐lowering treatment patterns and cardiovascular disease correlates in the same 9000‐person cohort [8, 19, 20, 21]. The 1486‐P conference abstract reported 9.6% simultaneous attainment [19], whereas the subsequent full article reported 5.0% [8]. Historical reporting denominators and analysis details were not identical, so these percentages should not be compared directly. The present study uses the same baseline data and adds BMI‐specific estimates of composite and component attainment, continuous BMI modeling and BMI‐stratified medication profiles. Its contribution is a focused secondary analysis of heterogeneity within this cohort, rather than evidence from an independent population (Table S1).

Our findings extend earlier Chinese and international data. In the nationwide 3B study conducted in 2010–2011, overweight and obese outpatients with Type 2 diabetes were less likely than those with normal BMI to reach all three targets, although that study used a blood‐pressure threshold of < 140/90 mmHg and did not account for center clustering or missing data [23]. Simultaneous control of glycemia, blood pressure, and LDL‐C remains uncommon across health systems [24, 25], and therapeutic inertia and physicians' underestimation of cardiovascular risk are recognized contributors [26]. The present findings place this pattern in a contemporary setting with substantial use of SGLT2 inhibitors and GLP‐1 receptor agonists.

Participants with obesity were younger and had shorter diabetes duration, yet had higher blood pressure and triglyceride levels and the lowest observed composite attainment, consistent with the obesity‐related hypertension and atherogenic dyslipidemia phenotype [11, 27]. Greater use of GLP‐1 receptor agonists and SGLT2 inhibitors at higher BMI is compatible with contemporary prescribing priorities that include weight management [28]. These cross‐sectional prescribing patterns do not estimate treatment effectiveness. Confounding by indication, disease severity, access to treatment, and previous treatment response can influence both current drug use and target attainment; the baseline data cannot separate these influences from drug effects.

Statin use was lower in obesity than overweight, whereas adjusted LDL‐C target attainment did not differ by BMI. This finding applies to the study threshold of < 2.6 mmol/L. Patients with established ASCVD or high cardiovascular risk generally require lower LDL‐C levels [6, 7], so meeting this threshold does not establish adequate lipid control for an individual patient. The uniform threshold permits a consistent comparison across BMI groups, but cannot quantify attainment of risk‐stratified goals; the information needed to assign these goals reliably was unavailable.

Only 99 participants (1.2%) had underweight. The pooled estimate for composite attainment was imprecise (OR 1.34, 95% CI 0.48–3.71), and the complete‐case estimate differed in direction. Low BMI can accompany β‐cell dysfunction, sarcopenia, undernutrition, or chronic comorbidity [9, 12, 16, 29], and has been associated with adverse outcomes in other populations with diabetes [15]. The present subgroup findings are exploratory and do not support a conclusion that underweight improves or worsens composite target attainment.

Continuous modeling showed a broadly linear association between BMI and composite attainment, with consistent findings under alternative knot numbers and removal of extreme BMI values. HbA1c and blood pressure attainment showed nonlinearity, whereas LDL‐C attainment showed neither an overall nor a nonlinear BMI association. The joint BMI–WHtR model was exploratory. Because BMI and WHtR capture overlapping dimensions of adiposity, collinearity limits interpretation of their mutually adjusted coefficients; this model does not establish which measure is more informative.

### 4.1 Clinical Implications

For adults with obesity, the clearest deficits in target attainment were in glycemia and blood pressure. Clinical review should therefore give particular attention to these two domains, alongside lipid management according to cardiovascular risk.

### 4.2 Strengths and Limitations

Strengths include the large multicenter cohort, explicit outcome definitions, and assessment of both categorical and continuous BMI. Multiple imputation, center‐clustered inference, and sensitivity analyses addressed missing data and variation across centers.

The cross‐sectional design does not establish temporal or causal relationships. Information on medication indications, dose, duration, adherence, and contraindications was unavailable. Among participants, 22.2% lacked at least one observed 3B component. Multiple imputation assumes missing at random conditional on included variables. BMI‐unavailable records differed from the analytic cohort, so selection bias remains possible despite a consistent selection‐weighted sensitivity analysis. The 61 center codes could not be mapped to the 60 hospitals. BMI does not distinguish fat from lean mass or capture normal‐weight adiposity [30, 31], and WHtR was unavailable for 14.8%. Center‐specific event counts were small, and secondary analyses used nominal *p* values. Hospital‐based recruitment limits generalizability.

## 5 Conclusions

In this hospital‐based cohort of Chinese adults with Type 2 diabetes, obesity was associated with lower odds of simultaneous 3B target attainment. Component‐specific associations were present for glycemic and blood‐pressure control, but not for LDL‐C attainment at the study threshold. Recorded use of GLP‐1 receptor agonists and SGLT2 inhibitors increased across BMI categories.

## Author Contributions

J.Y., W.Y., L.J., and X.C. conceived and designed the study. J.Y., W.Y., M.L., B.F., Y.L., Q.J., N.T., L.J., and X.C. acquired the data. J.Y., W.Y., and X.C. directly accessed and verified the underlying data. J.Y. and W.Y. performed the statistical analysis and drafted the manuscript. All authors interpreted the results, critically revised the manuscript, approved the final version, and accept responsibility for the work.

## Funding

This study was supported by the National Clinical Key Specialty Construction Program of China (2024; Department of Endocrinology, Peking University People's Hospital) and sponsored by AstraZeneca Investment (China) Co. Ltd. Financial support included participating‐site activities. The sponsor did not perform the present secondary statistical analysis, determine its interpretation, or make the decision to submit this manuscript. Participating investigators or sites received payments supporting clinical service, data collection, and study reporting. The sponsor did not perform the present secondary statistical analysis or determine its interpretation.

## Ethics Statement

The study was conducted in accordance with the Declaration of Helsinki and ICH‐GCP guidelines. Ethical approval was granted by the Ethics Committee of Peking University People's Hospital (approval number: 2023PHB063‐003) and endorsed by the institutional review boards of all participating sites.

## Consent

Written informed consent was obtained from all participants before enrollment.

## Conflicts of Interest

The authors declare no conflicts of interest.

## Supporting information


**Table S1:** Comparison with previous iCaReMe China reports.
**Table S2:** Comparison of BMI‐evaluable and BMI‐unavailable records.
**Table S3:** Missingness in the adult analytic cohort.
**Table S4:** Missingness by BMI category and across operational centers.
**Table S5:** Comparison of complete and incomplete 3B‐component records.
**Table S6:** Multiple‐imputation convergence and Monte Carlo precision.
**Table S7:** Observed composite 3B outcome status.
**Table S8:** Selected baseline covariates and target attainment by BMI category.
**Table S9:** Primary and sensitivity associations with the composite 3B target.
**Table S10:** Center‐code and selection‐weighted sensitivity analyses.
**Table S11:** Baseline medication use by BMI category.
**Table S12:** Adjusted associations with individual target components.
**Table S13:** Restricted cubic spline joint tests.
**Table S14:** Restricted cubic spline sensitivity analyses.
**Figure S1:** Multiple‐imputation chain means.
**Figure S2:** Multiple‐imputation chain variances.
**Figure S3:** Study flow.
**Figure S4:** Primary adjusted associations.
**Figure S5:** Adjusted restricted cubic spline associations.

## Data Availability

The de‐identified individual‐level data are not publicly available because of institutional and ethical restrictions. They may be obtained from the corresponding authors on reasonable request, subject to approval by the registry steering committee and participating institutions and applicable data‐use agreements. Analysis code and aggregate statistical diagnostics, including complete imputation traces, are available from the corresponding authors upon reasonable request.
