# Supplementary material for: Body Mass Index, Cardiometabolic Treatment Patterns and Multifactorial Risk‐Factor Control in Chinese Adults With Type 2 Diabetes: A Multicenter Cross‐Sectional Study
Source: J Diabetes. 2026 Nov 3;18(11):e70296. doi: 10.1111/1753-0407.70296 (PMC13731742; doi:10.1111/1753-0407.70296)
Supplement: Supplementary file 1 [file JDB-18-e70296-s001.docx]

**Supplementary Appendix 1**

*Body mass index, cardiometabolic treatment patterns and multifactorial risk-factor control in Chinese adults with type 2 diabetes: a multicenter cross-sectional study*

Supporting information for publication

# Contents

Supplementary Methods

Supplementary Table S1. Comparison with previous iCaReMe China reports

Supplementary Table S2. Comparison of BMI-evaluable and BMI-unavailable records

Supplementary Table S3. Missingness in the adult analytic cohort

Supplementary Table S4. Missingness by BMI category and across operational centers

Supplementary Table S5. Comparison of complete and incomplete 3B-component records

Supplementary Table S6. Multiple-imputation convergence and Monte Carlo precision

Supplementary Table S7. Observed composite 3B outcome status

Supplementary Table S8. Selected baseline covariates and target attainment by BMI category

Supplementary Table S9. Primary and sensitivity associations with the composite 3B target

Supplementary Table S10. Center-code and selection-weighted sensitivity analyses

Supplementary Table S11. Baseline medication use by BMI category

Supplementary Table S12. Adjusted associations with individual target components

Supplementary Table S13. Restricted cubic spline joint tests

Supplementary Table S14. Restricted cubic spline sensitivity analyses

Supplementary Figure S1. Multiple-imputation chain means

Supplementary Figure S2. Multiple-imputation chain variances

Supplementary Figure S3. Study flow

Supplementary Figure S4. Primary adjusted associations

Supplementary Figure S5. Adjusted restricted cubic spline associations

# **Supplementary Methods**

## 1. **Cohort and center identifier**

The frozen source extract contained 9,000 records recruited from 60 hospitals, as reported previously. We excluded 408 records without calculable BMI and two records with a recorded age below 18 years, yielding 8,590 adults. The source values were not altered. The analytic identifier contained 61 operational recruitment-center codes. Because the supplied files did not include a code-to-hospital crosswalk, the codes were retained as statistical clusters and were not interpreted as 61 hospitals. The smallest code (024) contained four adults; excluding it left 60 clusters and did not materially change estimates.

## 2. **Outcome construction**

The standardized 3B target required HbA1c <7.0%, systolic/diastolic blood pressure <130/80 mm Hg and LDL-C <2.6 mmol/L. In observed-data tables, records were classified as achieved only when all three components were observed and achieved, failed when at least one observed component failed, and indeterminate when at least one component was missing and none of the observed components failed. In each imputed dataset, component targets and the composite were derived passively from the imputed continuous measurements. Observed measurements were not overwritten.

## 3. **Vascular-history variables**

Standardized macrovascular history was coded as present when any of the registry fields for prior macrovascular disease or procedures was recorded as present, as absent when the “none of the above” field was ticked and no component was recorded as present, and as missing otherwise. Where a component was recorded as present but the “none of the above” field was also ticked, the positive component took precedence; this occurred in 884 records. Standardized microvascular history was coded in the same way from the registry fields for retinopathy, nephropathy and other microvascular disease, with 488 such conflicts. In the adult cohort, macrovascular history was present in 1,677, absent in 6,230 and missing in 683 participants; microvascular history was present in 1,891, absent in 5,638 and missing in 1,061. Absence of documentation was not treated as absence of disease, and the macrovascular variable was imputed alongside the other covariates.

## 4. **Multiple imputation**

Missing covariates and continuous outcome components were handled using chained-equations multiple imputation. Twenty independent completed datasets were generated in four batches of five using fixed random seeds, with 20 iterations per chain. Batch seeds were 20261831, 20262831, 20263831 and 20264831. Continuous variables used predictive mean matching with five donors and stochastic coefficient and residual-variance draws; binary variables used logistic-regression coefficient draws; unordered categorical variables used multinomial regression with Bayesian-bootstrap weights. The imputation model included all analysis variables, BMI spline terms, center identifier, medication indicators and auxiliary renal and metabolic markers. BMI, center identifier and originally observed values remained unchanged.

Diagnostics included iteration-by-iteration chain means and variances, R-hat summaries over all 20 and the final 10 iterations, warning logs, confirmation that observed values were unchanged, observed-versus-imputed distribution comparisons, range and blood-pressure consistency checks, and convergence of every downstream GEE fit. Across all 20 iterations, all mean and variance R-hat values were below 1.05. In the final 10 iterations the highest values were 1.063 for systolic-BP chain means and 1.059 for systolic-BP chain variances (diastolic BP 1.046 and 1.057, respectively), without visible directional drift. Primary-term Monte Carlo errors were 8.7%–10.7% of total standard errors. No conditional-model warnings occurred, all values passed range and consistency checks, and all pooled models converged.

## 5. **Regression models**

The primary population-averaged model was logistic GEE with exchangeable working correlation and robust sandwich standard errors, clustered by operational recruitment-center identifier. Covariates were age, sex, diabetes duration, smoking, alcohol use, education, physical activity and standardized macrovascular history; normal weight was the reference. GEE was selected for a population-average estimand. Estimates were combined using Rubin's rules with small-sample finite-cluster degrees of freedom. Primary-model working correlations ranged from 0.0325 to 0.0404 across imputations. Clustering was retained because measurement and practice patterns may be shared within centers.

## 6. **Sensitivity analyses**

Sensitivity analyses used: (1) complete cases with the same GEE; (2) an independence working correlation; (3) exclusion of code 024, the four-record operational center; (4) inverse-probability weighting for BMI evaluability, using observed demographics, available component measurements, medications, vascular history and center with weights truncated at the 1st/99th percentiles; (5) continuous BMI after trimming its 1st/99th percentiles; (6) a joint BMI and waist-to-height ratio model; (7) exploratory adjustment for insulin, metformin, SGLT2 inhibitor, GLP-1 receptor agonist, antihypertensive and statin use; and (8) two delta-adjusted missing-not-at-random scenarios, in which the imputed values of the originally missing outcome components were shifted before the targets were derived: the favorable scenario subtracted 0.5 percentage points from HbA1c, 5 mm Hg from systolic and 3 mm Hg from diastolic blood pressure and 0.3 mmol/L from LDL-C, and the unfavorable scenario added the same amounts. Observed values were not shifted. The complete-case analysis contained 6,181 participants, 289 composite events and 60 represented centers.

## 7. **Restricted cubic splines**

Continuous BMI was represented using restricted cubic splines with knots at 20.28, 24.17, 26.84 and 32.70 kg/m² (5th, 35th, 65th and 95th percentiles). BMI 24 kg/m² was the reference. D1 joint tests compared the spline with a model omitting BMI and with a linear BMI model. Composite-outcome sensitivity analyses used three knots at the 10th/50th/90th percentiles, five knots at the 5th/27.5th/50th/72.5th/95th percentiles, and the primary knots after trimming the 1st/99th percentiles. Plots span the 0.5th–99.5th percentiles and include a histogram strip (0.5 kg/m² bins) showing the observed BMI distribution.

## 8. **Software and reproducibility**

Imputation, GEE fitting, pooling, data validation, descriptive summaries and figures used Python 3.12 code (NumPy 2.3.5, pandas 2.2.3, SciPy 1.17.0 and scikit-learn 1.8.0). The implementation preserved the specified variables, stochastic predictive mean matching, model formulas, working correlations, robust sandwich variance and Rubin pooling. Complete-case validation against the archived implementation showed a maximum relative difference of 0.062% in BMI-category ORs. Source code, aggregate diagnostics, complete imputation traces, per-imputation model diagnostics and range checks are available from the corresponding authors upon reasonable request.

**Supplementary Table S1. Comparison with previous iCaReMe China reports**

| **Report** | **Design** | **Population** | **Distinct contribution** |
| --- | --- | --- | --- |
| Cai et al., Advances in Therapy, 2025 | Registry design | Planned approximately 19,000 across four disease cohorts and 110 sites | Rationale, governance, eligibility and planned outcomes; no BMI-specific results |
| Yang et al., Diabetes 2025; abstract 1486-P | Conference abstract | 9,000 adults with type 2 diabetes from 60 hospitals | Overall clinical profile and unadjusted national target attainment |
| Wu et al., Diabetes 2025; abstract 848-P | Conference abstract | 9,000 adults with type 2 diabetes from 60 hospitals | Overall glucose-lowering treatment patterns, including combination therapy and complication strata; no BMI stratification |
| Cai et al., Diabetes 2025; abstract 440-P | Conference abstract | 9,000 adults with type 2 diabetes from 60 sites | Factors associated with cardiovascular disease; no BMI-specific 3B analysis |
| Yang et al., Diabetes, Obesity and Metabolism, 2026 | Full aggregate report | 9,000 adults with type 2 diabetes from 60 hospitals | Overall cardiovascular risk-factor control and broad correlates |
| Present study | BMI-focused secondary analysis | 8,590 adults with calculable BMI from the same 60-hospital cohort | BMI-specific treatment patterns; standardized 3B coding; MI; operational-center-clustered GEE; formal spline, WHtR, complete-case and MNAR analyses |

The prior aggregate report described recruitment from 60 hospitals. Operational database identifiers were used for statistical clustering and were not interpreted as a revised hospital count.

**Supplementary Table S2. Comparison of adults with evaluable BMI and records excluded because height or weight was unavailable**

Values are mean (SD); median (interquartile range) or n/N (%). Standardized differences describe imbalance and are emphasized over P values. Positive values indicate higher values or prevalence in the BMI-evaluable group.

| **Characteristic** | **BMI evaluable (N=8,590)** | **BMI unavailable (N=408)** | **Standardized difference** |
| --- | --- | --- | --- |
| Age, years | 54.16 (12.25); 55.00 (46.00–63.00) | 53.63 (11.45); 55.00 (47.00–61.00) | 0.045 |
| Diabetes duration, years | 7.50 (7.41); 5.42 (0.95–11.54) | 5.86 (6.36); 3.67 (0.54–9.51) | 0.237 |
| HbA1c, % | 8.56 (2.24); 8.00 (6.80–10.00) | 7.96 (2.13); 7.30 (6.50–9.00) | 0.277 |
| Systolic BP, mm Hg | 131.11 (16.25); 130 (120–140) | 135.18 (15.24); 135 (124–143) | −0.258 |
| LDL-C, mmol/L | 2.83 (0.99); 2.77 (2.13–3.44) | 2.80 (0.96); 2.75 (2.12–3.43) | 0.034 |
| Statin use | 2,874/8,590 (33.5%) | 89/408 (21.8%) | 0.263 |
| Antihypertensive use | 3,474/8,590 (40.4%) | 116/408 (28.4%) | 0.255 |
| Macrovascular history | 1,677/7,907 (21.2%) | 52/378 (13.8%) | 0.197 |
| HbA1c target achieved | 2,208/7,720 (28.6%) | 126/327 (38.5%) | −0.211 |

**Supplementary Table S3. Missingness in the adult analytic cohort (N=8,590)**

| **Domain** | **Variable** | **Missing** | **Missing (%)** |
| --- | --- | --- | --- |
| Outcome component | HbA1c | 870 | 10.13 |
| Outcome component | Systolic BP | 298 | 3.47 |
| Outcome component | Diastolic BP | 299 | 3.48 |
| Outcome component | LDL-C | 1,398 | 16.27 |
| Outcome summary | At least one unavailable 3B component | 1,907 | 22.20 |
| Covariate | Diabetes duration | 198 | 2.31 |
| Covariate | Smoking | 63 | 0.73 |
| Covariate | Alcohol use | 218 | 2.54 |
| Covariate | Education | 679 | 7.90 |
| Covariate | Physical activity | 1,061 | 12.35 |
| Covariate | Macrovascular history | 683 | 7.95 |
| Sensitivity variable | Waist-to-height ratio | 1,273 | 14.82 |

BMI and age were complete after cohort selection. Missing outcome components were imputed on their continuous scales; target indicators were then derived passively.

**Supplementary Table S4. Missingness by BMI category and across operational centers**

| **Variable** | **Overall n (%)** | **Underweight %** | **Normal %** | **Overweight %** | **Obesity %** | **Center median (IQR); range, %** |
| --- | --- | --- | --- | --- | --- | --- |
| HbA1c | 870 (10.1) | 11.1 | 9.9 | 10.5 | 9.8 | 5.6 (1.6–10.2); 0.0–54.7 |
| Systolic BP | 298 (3.5) | 6.1 | 3.8 | 3.4 | 3.0 | 1.0 (0.0–3.4); 0.0–55.1 |
| Diastolic BP | 299 (3.5) | 6.1 | 3.8 | 3.5 | 3.0 | 1.0 (0.0–3.4); 0.0–55.1 |
| LDL-C | 1,398 (16.3) | 20.2 | 16.8 | 16.6 | 14.9 | 9.7 (3.0–22.4); 0.0–66.0 |
| Diabetes duration | 198 (2.3) | 2.0 | 2.8 | 2.2 | 1.9 | 0.0 (0.0–1.5); 0.0–43.6 |
| Smoking | 63 (0.7) | 1.0 | 0.5 | 0.7 | 1.1 | 0.0 (0.0–0.8); 0.0–8.8 |
| Alcohol use | 218 (2.5) | 5.1 | 2.6 | 2.3 | 2.7 | 0.0 (0.0–2.4); 0.0–18.9 |
| Education | 679 (7.9) | 5.1 | 6.9 | 7.9 | 9.2 | 0.7 (0.0–4.7); 0.0–84.1 |
| Physical activity | 1,061 (12.4) | 15.2 | 11.8 | 12.1 | 13.4 | 2.0 (0.5–14.3); 0.0–100.0 |
| Macrovascular history | 683 (8.0) | 6.1 | 9.1 | 7.1 | 8.0 | 3.5 (0.8–6.1); 0.0–89.1 |
| Waist-to-height ratio | 1,273 (14.8) | 24.2 | 16.0 | 14.4 | 13.6 | 2.3 (0.0–25.3); 0.0–93.9 |

BMI-category percentages use the corresponding adult BMI group. Center summaries describe the distribution of center-specific missing percentages across 61 operational codes; extreme center values are shown transparently and center was included in imputation.

**Supplementary Table S5. Comparison of complete and incomplete 3B-component records**

| **Characteristic** | **All components observed (N=6,683)** | **≥1 component unavailable (N=1,907)** | **Standardized difference** |
| --- | --- | --- | --- |
| Age, years | 53.88 (12.21); n=6,683 | 55.13 (12.36); n=1,907 | -0.101 |
| Male sex | 4,160/6,683 (62.2%) | 1,073/1,907 (56.3%) | 0.122 |
| BMI, kg/m2 | 25.90 (3.91); n=6,683 | 25.70 (3.89); n=1,907 | 0.051 |
| Diabetes duration, years | 7.47 (7.41); n=6,526 | 7.61 (7.39); n=1,866 | -0.019 |
| HbA1c, % | 8.60 (2.24); n=6,683 | 8.30 (2.24); n=1,037 | 0.134 |
| Systolic BP, mm Hg | 131.44 (16.45); n=6,683 | 129.76 (15.34); n=1,609 | 0.105 |
| LDL-C, mmol/L | 2.84 (1.00); n=6,683 | 2.71 (0.92); n=509 | 0.138 |
| Macrovascular history | 1,310/6,233 (21.0%) | 367/1,674 (21.9%) | -0.022 |
| Insulin use | 2,792/6,683 (41.8%) | 680/1,907 (35.7%) | 0.126 |
| SGLT2 inhibitor use | 2,705/6,683 (40.5%) | 724/1,907 (38.0%) | 0.051 |
| GLP-1 receptor agonist use | 1,142/6,683 (17.1%) | 283/1,907 (14.8%) | 0.061 |
| Antihypertensive use | 2,706/6,683 (40.5%) | 768/1,907 (40.3%) | 0.004 |
| Statin use | 2,363/6,683 (35.4%) | 511/1,907 (26.8%) | 0.186 |

Continuous values are mean (SD) with observed n; categorical values are n/N (%). Positive standardized differences indicate higher values or prevalence in complete records. Component-variable summaries in incomplete records use available observations only.

**Supplementary Table S6. Multiple-imputation convergence and Monte Carlo precision**

| **Imputed variable** | **R-hat, chain means (all 20 iterations)** | **R-hat, chain variances (all 20 iterations)** |
| --- | --- | --- |
| duration | 0.991 | 0.992 |
| smoking | 1.008 | 0.999 |
| alcohol | 0.986 | 0.998 |
| education | 0.994 | 1.002 |
| activity | 0.998 | 0.999 |
| macrovascular | 0.988 | 0.987 |
| hba1c | 1.025 | 1.015 |
| sbp | 1.017 | 1.012 |
| dbp | 1.018 | 1.035 |
| ldlc | 1.021 | 0.996 |
| whtr_sensitivity | 0.998 | 1.000 |
| fasting_glucose | 0.988 | 0.994 |

All full-chain R-hat values were below 1.05. Final-10-iteration summaries are reported in the Supplementary Methods; complete trace data are available from the corresponding authors upon reasonable request. Primary-term precision: Underweight: FMI 0.275, MCSE/SE 0.107; Overweight: FMI 0.192, MCSE/SE 0.087; Obesity: FMI 0.195, MCSE/SE 0.087.

**Supplementary Table S7. Observed composite 3B outcome status**

| **BMI category** | **Total** | **Complete: achieved** | **Complete: failed** | **Incomplete: known failed** | **Indeterminate** |
| --- | --- | --- | --- | --- | --- |
| Underweight | 99 | 3 | 69 | 16 | 11 |
| Normal weight | 2,725 | 140 | 1,958 | 485 | 142 |
| Overweight | 3,663 | 139 | 2,705 | 680 | 139 |
| Obesity | 2,103 | 50 | 1,619 | 376 | 58 |
| Total | 8,590 | 332 | 6,351 | 1,557 | 350 |

An incomplete record was a known failure if at least one observed component failed. It was indeterminate only when no observed component failed. The primary inferential analysis multiply imputed component measurements instead of assigning indeterminate records to failure.

**Supplementary Table S8. Selected baseline covariates and target attainment by BMI category**

| **Characteristic** | **Underweight (n=99)** | **Normal weight (n=2,725)** | **Overweight (n=3,663)** | **Obesity (n=2,103)** |
| --- | --- | --- | --- | --- |
| Age, years | 60.0 (51.0–66.5) | 58.0 (50.0–65.0) | 56.0 (47.0–63.0) | 50.0 (39.0–59.0) |
| Male sex, n/N (%) | 49/99 (49.5%) | 1489/2725 (54.6%) | 2354/3663 (64.3%) | 1341/2103 (63.8%) |
| Diabetes duration, years | 5.5 (0.6–10.7) | 6.7 (1.6–13.1) | 5.8 (1.2–12.1) | 3.4 (0.3–9.6) |
| Macrovascular history, n/N (%) | 25/93 (26.9%) | 504/2477 (20.3%) | 789/3403 (23.2%) | 359/1934 (18.6%) |
| Microvascular history, n/N (%) | 29/81 (35.8%) | 584/2382 (24.5%) | 834/3227 (25.8%) | 444/1839 (24.1%) |
| Hypertension history, n/N (%) | 29/96 (30.2%) | 939/2644 (35.5%) | 1609/3553 (45.3%) | 996/2027 (49.1%) |
| HbA1c target, n/N (%) | 19/88 (21.6%) | 810/2456 (33.0%) | 925/3280 (28.2%) | 454/1896 (23.9%) |
| Blood-pressure target, n/N (%) | 45/93 (48.4%) | 938/2622 (35.8%) | 915/3537 (25.9%) | 408/2040 (20.0%) |
| LDL-C target, n/N (%) | 36/79 (45.6%) | 1022/2267 (45.1%) | 1311/3056 (42.9%) | 722/1790 (40.3%) |
| Composite 3B target, n/N (%) | 3/72 (4.2%) | 140/2098 (6.7%) | 139/2844 (4.9%) | 50/1669 (3.0%) |

Values are median (interquartile range) or n/N (%). Denominators reflect observed availability. Standardized vascular histories use the validated composite variables rather than treating unavailable source fields as absence.

**Supplementary Table S9. Primary and sensitivity associations with the composite 3B target**

| **Analysis** | **BMI category** | **Adjusted OR (95% CI)** | **P value** | **Sample** |
| --- | --- | --- | --- | --- |
| Primary MI GEE | Underweight | 1.34 (0.48–3.71) | 0.568 | 8,590 |
| Primary MI GEE | Overweight | 0.78 (0.59–1.04) | 0.090 | 8,590 |
| Primary MI GEE | Obesity | 0.55 (0.39–0.78) | 0.001 | 8,590 |
| Complete-case GEE | Underweight | 0.55 (0.14–2.08) | 0.368 | 6,181 |
| Complete-case GEE | Overweight | 0.79 (0.58–1.07) | 0.122 | 6,181 |
| Complete-case GEE | Obesity | 0.50 (0.36–0.70) | <0.001 | 6,181 |
| Independence-correlation MI GEE | Underweight | 1.29 (0.50–3.31) | 0.586 | 8,590 |
| Independence-correlation MI GEE | Overweight | 0.75 (0.58–0.96) | 0.026 | 8,590 |
| Independence-correlation MI GEE | Obesity | 0.53 (0.36–0.77) | 0.001 | 8,590 |
| Favorable MNAR delta | Underweight | 1.25 (0.48–3.23) | 0.642 | 8,590 |
| Favorable MNAR delta | Overweight | 0.79 (0.60–1.03) | 0.078 | 8,590 |
| Favorable MNAR delta | Obesity | 0.56 (0.40–0.78) | <0.001 | 8,590 |
| Unfavorable MNAR delta | Underweight | 1.29 (0.46–3.63) | 0.617 | 8,590 |
| Unfavorable MNAR delta | Overweight | 0.81 (0.60–1.09) | 0.154 | 8,590 |
| Unfavorable MNAR delta | Obesity | 0.59 (0.41–0.84) | 0.004 | 8,590 |
| Medication-adjusted exploratory | Underweight | 1.68 (0.59–4.77) | 0.322 | 8,590 |
| Medication-adjusted exploratory | Overweight | 0.82 (0.61–1.10) | 0.182 | 8,590 |
| Medication-adjusted exploratory | Obesity | 0.60 (0.43–0.85) | 0.005 | 8,590 |

All models used normal weight as the reference and adjusted for age, sex, diabetes duration, smoking, alcohol use, education, physical activity and macrovascular history. MNAR, missing not at random; MI, multiple imputation; GEE, generalized estimating equations.

**Supplementary Table S10. Center-code and selection-weighted sensitivity analyses**

| **Analysis** | **BMI category** | **Adjusted OR (95% CI)** | **P value** |
| --- | --- | --- | --- |
| Exclude four-record center 024 (exchangeable GEE) | Underweight | 1.34 (0.48–3.70) | 0.568 |
| Exclude four-record center 024 (exchangeable GEE) | Overweight | 0.78 (0.59–1.04) | 0.090 |
| Exclude four-record center 024 (exchangeable GEE) | Obesity | 0.55 (0.39–0.78) | 0.001 |
| BMI-selection weighted (independence GEE) | Underweight | 1.29 (0.50–3.32) | 0.588 |
| BMI-selection weighted (independence GEE) | Overweight | 0.74 (0.57–0.96) | 0.022 |
| BMI-selection weighted (independence GEE) | Obesity | 0.53 (0.36–0.77) | 0.001 |

Normal weight was the reference. The center-code analysis removed code 024 (n=4), leaving 60 clusters. The selection model used all 8,998 adult records, including 408 without calculable BMI; stabilized weights were truncated at the 1st and 99th percentiles (range 0.956–1.651; effective n=8,527). The weighted model used an independence working correlation and cluster-robust variance.

**Supplementary Table S11. Baseline medication use by BMI category**

| **Medication** | **Underweight** | **Normal weight** | **Overweight** | **Obesity** |
| --- | --- | --- | --- | --- |
| Metformin | 35/99 (35.4%) | 1380/2725 (50.6%) | 2101/3663 (57.4%) | 1312/2103 (62.4%) |
| Insulin | 68/99 (68.7%) | 1177/2725 (43.2%) | 1476/3663 (40.3%) | 751/2103 (35.7%) |
| SGLT2 inhibitor | 16/99 (16.2%) | 908/2725 (33.3%) | 1550/3663 (42.3%) | 955/2103 (45.4%) |
| GLP-1 receptor agonist | 5/99 (5.1%) | 159/2725 (5.8%) | 586/3663 (16.0%) | 675/2103 (32.1%) |
| Any antihypertensive | 24/99 (24.2%) | 897/2725 (32.9%) | 1568/3663 (42.8%) | 985/2103 (46.8%) |
| Angiotensin receptor blocker | 8/99 (8.1%) | 358/2725 (13.1%) | 692/3663 (18.9%) | 476/2103 (22.6%) |
| ACE inhibitor | 1/99 (1.0%) | 46/2725 (1.7%) | 82/3663 (2.2%) | 45/2103 (2.1%) |
| Statin | 29/99 (29.3%) | 913/2725 (33.5%) | 1331/3663 (36.3%) | 601/2103 (28.6%) |
| Any antiplatelet | 17/99 (17.2%) | 367/2725 (13.5%) | 600/3663 (16.4%) | 232/2103 (11.0%) |
| Any anticoagulant | 1/99 (1.0%) | 11/2725 (0.4%) | 15/3663 (0.4%) | 7/2103 (0.3%) |

Medication indicators describe recorded current use at baseline. The dataset did not provide a complete indication-eligible denominator, dose, duration, adherence, contraindication or affordability information; no treatment-effect inference was made.

**Supplementary Table S12. Adjusted associations with individual target components in the primary MI GEE**

| **Outcome** | **BMI category** | **Adjusted OR (95% CI)** | **P value** |
| --- | --- | --- | --- |
| HbA1c <7.0% | Underweight | 0.64 (0.37–1.11) | 0.108 |
| HbA1c <7.0% | Overweight | 0.85 (0.78–0.94) | 0.001 |
| HbA1c <7.0% | Obesity | 0.72 (0.61–0.85) | <0.001 |
| BP <130/80 mm Hg | Underweight | 1.61 (1.10–2.36) | 0.015 |
| BP <130/80 mm Hg | Overweight | 0.66 (0.59–0.74) | <0.001 |
| BP <130/80 mm Hg | Obesity | 0.47 (0.40–0.55) | <0.001 |
| LDL-C <2.6 mmol/L | Underweight | 1.06 (0.67–1.67) | 0.802 |
| LDL-C <2.6 mmol/L | Overweight | 0.96 (0.85–1.08) | 0.499 |
| LDL-C <2.6 mmol/L | Obesity | 1.05 (0.91–1.22) | 0.486 |

Normal weight was the reference. Covariates and pooling were identical to the primary composite model.

**Supplementary Table S13. Restricted cubic spline joint tests**

| **Outcome** | **P overall** | **P nonlinear** | **Interpretation** |
| --- | --- | --- | --- |
| Composite 3B target | <0.001 | 0.904 | Overall association; no evidence of nonlinearity |
| HbA1c target | <0.001 | 0.001 | Overall and nonlinear association |
| Blood-pressure target | <0.001 | 0.021 | Overall and nonlinear association |
| LDL-C target | 0.556 | 0.382 | No evidence of overall or nonlinear association |

D1 tests combined evidence across 20 imputed datasets. The overall test compared the spline model with a model omitting BMI; the nonlinearity test compared the spline with a linear BMI term.

**Supplementary Table S14. Restricted cubic spline sensitivity analyses for the composite 3B target**

| **Analysis** | **N** | **BMI knots, kg/m²** | **P overall** | **P nonlinear** |
| --- | --- | --- | --- | --- |
| 3 knots: 10th/50th/90th percentiles | 8,590 | 21.45; 25.39; 30.80 | <0.001 | 0.751 |
| 5 knots: 5th/27.5th/50th/72.5th/95th | 8,590 | 20.28; 23.49; 25.39; 27.68; 32.70 | <0.001 | 0.851 |
| Primary 4 knots; trim 1st/99th percentiles | 8,419 | 20.28; 24.17; 26.84; 32.70 | <0.001 | 0.680 |

D1 tests were pooled across 20 imputed datasets. All analyses adjusted for the primary covariates and used center-clustered exchangeable-correlation GEE.

**Supplementary Figure S1. Multiple-imputation chain means**

Iteration-by-iteration means of imputed values across 20 independent chains. Panels use variable-specific scales.

**Supplementary Figure S2. Multiple-imputation chain variances**

Iteration-by-iteration variances of imputed values across 20 independent chains. Panels use variable-specific scales.

**Supplementary Figure S3. Study flow**

Derivation of the adult BMI analytic cohort from the 9,000 baseline records of the iCaReMe China type 2 diabetes cohort and its distribution across Chinese BMI categories. Operational center codes are database identifiers used for statistical clustering.

**Supplementary Figure S4. Primary adjusted associations of BMI categories with the composite target and its individual components**

Pooled exchangeable-correlation logistic GEE across 20 imputed datasets, clustered by operational recruitment-center identifier; normal weight was the reference; covariates as in the primary model. Estimates correspond to Supplementary Table S9 (primary rows) and Table S12.

**Supplementary Figure S5. Adjusted restricted cubic spline associations**

Odds ratios (log scale) are relative to BMI 24 kg/m² (vertical dotted line); shaded bands are pointwise 95% confidence intervals; triangles mark the knots at 20.28, 24.17, 26.84 and 32.70 kg/m²; curves span the 0.5th–99.5th BMI percentiles. Joint tests are reported in Table S13; the same curves with the observed BMI distribution appear as Figure 4 in the main text.
